# Editorial: Spatial and metabolic dimensions of immunosuppression in the solid tumor microenvironment

**DOI:** 10.3389/fimmu.2026.2019979

**Published:** 2026-10-26

**Authors:** Weiteng Lin, Yue Tian, Surendra K. Shukla, Chunbo He, Chenshen Huang

**Affiliations:** 1 Fuzhou University Affiliated Provincial Hospital, School of Medicine, Fuzhou University, Fuzhou, China; 2 Shengli Clinical Medical College of Fujian Medical University, Fuzhou, China; 3 Fujian Medical University Union Hospital, Fuzhou, China; 4 Department of Oncology Science, University of Oklahoma College of Medicine, University of Oklahoma Health Sciences, Oklahoma City, OK, United States

**Keywords:** immunosuppression, machine learning, metabolic reprogramming, spatial niches, tumor microenvironment

## Introduction

Immunosuppression in solid tumors is not governed by a single cell type or signaling pathway. Instead, it arises from interactions among tumor cells, immune cells, and stromal cells within specific microenvironmental contexts (1, 2). The functions of cell populations such as regulatory T cells (Tregs), tumor-associated macrophages (TAMs), and cancer-associated fibroblasts (CAFs) are jointly shaped by their spatial neighborhoods and local metabolic conditions (3, 4). The net impact of these cell populations is context dependent. They may promote tumor immune evasion, but may also restrain harmful inflammation or support antitumor immunity (2, 5, 6). Based on this understanding, this Research Topic brings together nine original research articles and four reviews spanning multiple levels, including spatial effects, metabolic niches, and clinical implications, collectively advancing our understanding of the multiscale features of immunosuppression in solid tumors.

## Spatial effects shape immune functional states

Cellular location and intercellular interactions are important determinants of immune functional states (3). Cai et al. integrated spatial transcriptomic data across multiple cancer types and found that COL11A1^+^ CAFs tend to accumulate at tumor boundaries and are associated with the reduced signatures of activated cytotoxic T cells and B cells, suggesting that they may participate in establishing boundaries that restrict immune-cell entry into tumors. Using multiplex immunofluorescence, Sun et al. found in non-small cell lung cancer that a spatial neighborhood composed of BAG3^+^IFITM2^+^ CAFs and BAG3^+^CD8^+^ T cells was associated with resistance to neoadjuvant chemoimmunotherapy. Notably, this configuration represents a setting in which CD8^+^ T cells are present but embedded within a specific CAF-associated stromal neighborhood. However, spatial proximity does not necessarily equate to functional interaction. For example, MUC16 can inhibit conjugation between NK cells and ovarian cancer cells and the formation of effective immune synapses (7). Furthermore, spatial organization is not static. In their review of prostate cancer, Xue et al. proposed a framework of tumor–immune spatiotemporal co-evolution and discussed how tumor progression and therapy-imposed selective pressures can continuously alter tumor clones, stromal architecture, and immune composition. Therefore, future spatial maps need to move beyond simply describing cell locations toward resolving dynamic functional niches jointly constituted by cellular states, tissue architecture, and intercellular interactions.

## Metabolic niches shape immune functional states

Spatial organization and metabolic states are not independent of one another (4, 8). Spatial transcriptomic analyses by Cai et al. also identified co-localization of COL11A1^+^ CAF and lipid-associated macrophage signatures, suggesting a possible link between tissue organization and metabolism-associated cellular states. Factors such as abnormal vasculature, hypoxia, and nutrient competition create regional heterogeneity in nutrients and metabolites and selectively support distinct cellular states (9–11). In microsatellite-stable gastric cancer, Lin et al. identified a lactylation-related gene signature that was associated with PDGFRA^+^ and CA9^+^ CAF states, hypoxia and extracellular matrix remodeling, and computationally predicted immune exclusion and dysfunction. Zheng further summarized amino acid, lipid, and glucose metabolic reprogramming in osteosarcoma–macrophage interactions, illustrating that metabolites can participate in TAM reprogramming, T-cell suppression, pre-metastatic niche formation, and treatment resistance. Liu further integrated lactate and acidosis, the hypoxia–adenosine axis, tricarboxylic acid-cycle metabolites, and mitochondrial damage signals as metabolic pressures that affect the adaptive capacity of different immune cells, and proposed the concept of “immune sorting,” whereby the microenvironment does not uniformly suppress all immune cells but selectively favors immune-cell states capable of remaining functional under the corresponding metabolic pressures. Meanwhile, Huang et al. used transcriptome-based metabolic modeling to infer increased glycolysis, fatty acid oxidation, tricarboxylic acid-cycle activity, and amino acid metabolism in high R-loop prognostic index (RLPI) glioma cells, alongside computational associations with immune infiltration and immunosuppressive signaling. Using genetic and Mendelian randomization analyses, Wu et al. further linked brain microstructure and cross-compartment metabolic features to glioma susceptibility, extending the scale at which spatial–metabolic interactions are considered. These studies suggest that metabolic reprogramming can generate local immune selection pressures and may also interact with tissue environments at larger scales.

## Context dependence of immune regulation and clinical implications

The immunological significance of cells or molecules within the tumor microenvironment cannot be defined independently of their specific context. Beyond previous studies on IL18BP (12, 13), the review by Huang et al. provides a representative example: by limiting receptor-accessible free IL-18, IL18BP can attenuate NK-cell- and T-cell-mediated antitumor responses; when IL-18 participates in chronic inflammation, immune tolerance, or metastatic adaptation, its neutralization may produce different biological consequences. Zhao et al. showed that extracellular vesicles from coal dust-stimulated macrophages promoted thyroid cancer cell proliferation and invasion *in vitro* and tumor growth in xenograft models, highlighting the context-dependent effects of macrophage-mediated communication on tumor progression. This context dependence also suggests that we need to shift from seeking universal single immune or metabolic biomarkers toward identifying the current immune ecosystem state of a tumor and its dominant limiting factors (14, 15). Sun et al. found that the BAG3^+^ CAF–T-cell neighborhood was associated with pathological non-response to neoadjuvant chemoimmunotherapy and showed greater discriminatory performance than either BAG3^+^IFITM2^+^ CAFs or BAG3^+^CD8^+^ T cells alone, suggesting that spatial relationships may provide information beyond individual cell populations. Peng et al. further showed that a Treg-rich state could be indirectly predicted by computational models based on routine H&E histomorphology, demonstrating the potential to extract information encoded in tissue morphology from whole-slide images and further translate complex microenvironmental information into accessible indicators (16).

These observations further underscore the practical need for clinically meaningful stratification. In retrospective analyses, the high RLPI defined by Huang et al. was associated with poorer responses to radiotherapy and temozolomide in lower-grade glioma, alongside increased computationally estimated immune-cell infiltration and immune-checkpoint gene programs. By contrast, a higher index was associated with more favorable responses to immune checkpoint inhibitors in independent cohorts of other cancer types. These findings suggest that different microenvironmental states may expose distinct therapeutic vulnerabilities. Complementing this molecular-stratification perspective, Yang et al. developed a five-gene parthanatos-related prognostic model in lung adenocarcinoma. The resulting risk groups differed in computationally estimated immune-cell infiltration and immune-checkpoint gene expression. The retrospective gastric cancer cohort study by Li et al. further illustrated, at the level of clinical endpoints, the importance of accurately identifying patients with potential non-response before treatment. Meanwhile, the reviews by Xue and Liu integrated evidence suggesting that treatment pressure, while eliminating sensitive cells, may simultaneously select or reshape new metabolic, stromal, and immune states. Therefore, future clinical translation should move beyond single-marker stratification toward dynamic biomarkers based on spatial effects, metabolic state, and intercellular interactions.

## Conclusion

This Research Topic demonstrates the multiscale features of immunosuppression in solid tumors from multiple perspectives, including spatial effects, metabolic adaptation, and clinical implications. These studies emphasize that the net effect of immune regulation is jointly influenced by cellular states, spatial neighborhoods, and metabolic resources. Viewed in the broader context of existing mechanistic evidence, these contributions offer complementary perspectives on the interplay among these dimensions. Spatial effects may shape local metabolic pressures, while metabolic reprogramming may, in turn, reshape cellular states and spatial interactions. This dynamic interplay might influence the context-dependent balance between immunosuppression and antitumor immunity, with implications for therapeutic responses (**Figure 1**) (17–19). The key challenge ahead is to further determine how specific microenvironmental conditions alter the net impact of immune cells and to construct spatially resolved, cross-scale maps of tumor–immune ecosystems. Ultimately, translating these findings into stratification tools and selective intervention strategies could help advance precision cancer therapy.

**Figure 1:**
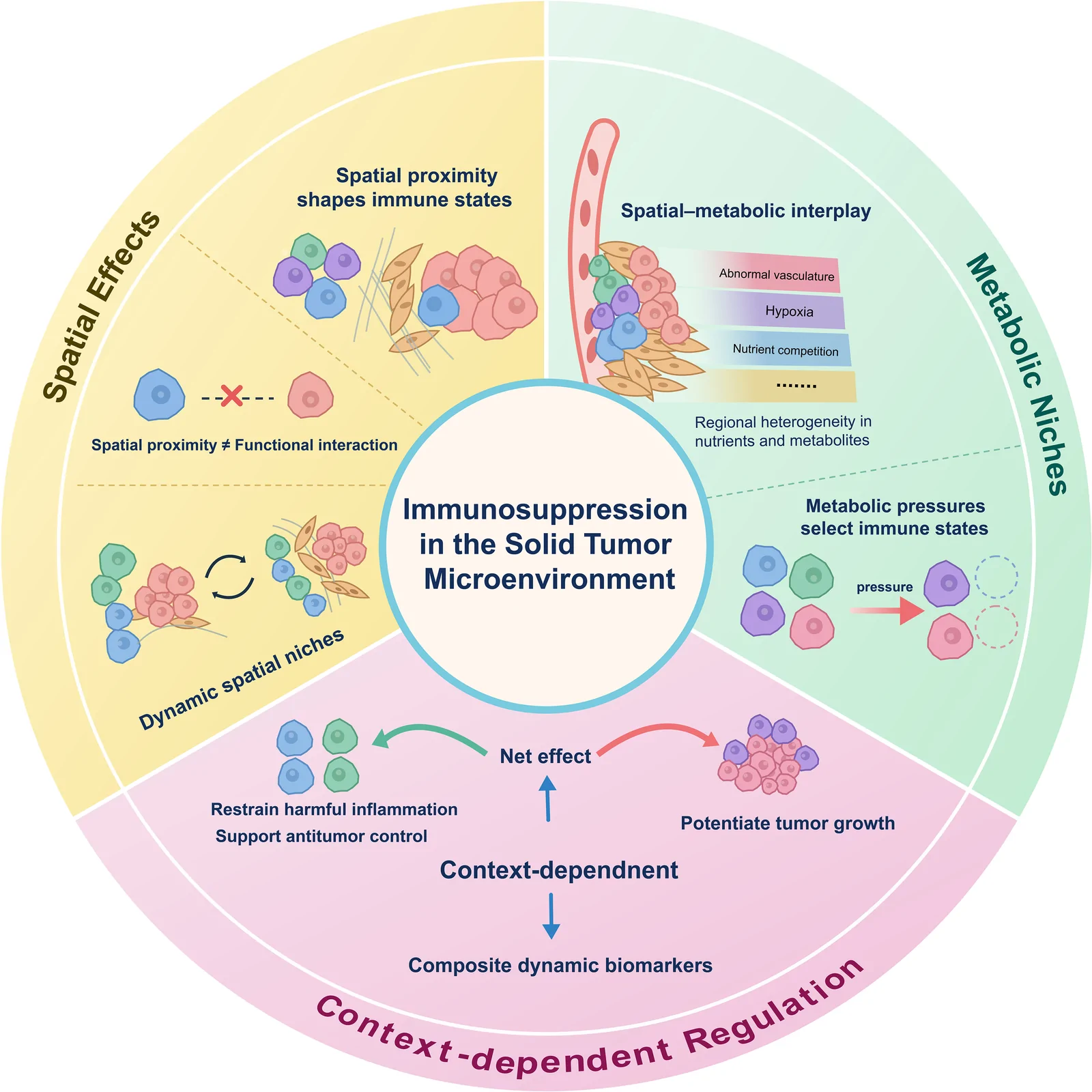
Spatial, metabolic, and context-dependent dimensions of immunosuppression in the solid tumor microenvironment.
